# A Fast and Simple Qualitative Method for Screening Oleaginous Yeasts on Agar

**DOI:** 10.1155/2018/5325804

**Published:** 2018-07-29

**Authors:** Xochitl Niehus, Leticia Casas-Godoy, Marcos Vargas-Sánchez, Georgina Sandoval

**Affiliations:** ^1^Centro de Investigación y Asistencia en Tecnología y Diseño del Estado de Jalisco A.C. (CIATEJ), 800 Normalistas Av., 44270 Guadalajara, JAL, Mexico; ^2^Cátedras CONACYT- Centro de Investigación y Asistencia en Tecnología y Diseño del Estado de Jalisco A.C. (CIATEJ), 800 Normalistas Av., 44270 Guadalajara, JAL, Mexico

## Abstract

Finding new oleaginous yeasts is of great interest due to their many important applications. Currently available screening procedures are time-consuming, and most of these require liquid cultures. In this work, a new, fast, economical, and simple qualitative method for screening oleaginous yeasts was developed. The fluorescent dye, Rhodamine B, was selected because its fluorescence is directly correlated to lipid content, and no additional steps or special equipment are needed. This method only requires growing the yeasts on dyed agar plates. Under visible light, it is easy to observe that nonpigmented oleaginous yeasts become colored, whereas non-oleaginous yeasts remain uncolored. The developed method is also useful for improving medium composition in specific applications. Moreover, it was also adapted to use alternative carbon sources, such as lignocellulosic materials and glycerol. The developed method was applied to screen 124 recently isolated nonpigmented yeasts on three different carbon sources, namely, glucose, glycerol, and agave bagasse hydrolysate. Five strains were selected as good lipid producers on all tested carbon sources and accumulated over 48% lipids. Furthermore, the assay was adapted to screen reddish-pigmented yeasts. Considering all the above, the developed method has a wide range of applications in the field of microbial oils.

## 1. Introduction

Microbial oils or single cell oils are defined as the oils produced by oleaginous microorganisms. These microorganisms are able to accumulate more than 20% of their dry cell weight (DCW) as lipids in the form of droplets inside the cells. This accumulation is mainly due to an excess of the carbon (C) source and limited availability of another nutrient, such as nitrogen (N) [1, 2]. Lipids from yeasts are mainly triacylglycerols, which can be compared, in terms of their chemical composition, to lipids obtained from plant oilseeds (vegetable oils). Furthermore, yeasts can use a wide range of nutrient sources, including industrial wastes, which could reduce production costs. Metabolic engineering is being carried out to obtain cells with higher lipid contents and thus obtain a higher final lipid yield [2, 3]. In these procedures, as well as in the isolation and search for new oleaginous yeasts, the selection of the colonies with the highest lipid production is desirable. Oils from yeasts have many applications in the food, pharmaceutical and biofuel industries. Among them, their use as alternative raw materials for biofuels production, such as biodiesel, stands out [2, 4]. Indeed, the common raw materials for biodiesel are vegetable oils, which represent approximately 88% of the production costs [5] and generate polemics regarding the usage of edible oils and arable land for biofuel production. Oils from yeasts also present the advantage of having better yields per area in a notably shorter production time compared with vegetable oils.

Therefore, the search for new oleaginous yeasts is of great interest. Although some qualitative and quantitative screening methods have been developed, they are very laborious and require liquid cultures, which are time- and resource-consuming, or entail the use of hazardous compounds.

Among the available quantitative methods for the screening of oleaginous yeasts, the method described by Kimura et al. [6] and its modifications [7] are the most commonly used. These methods require liquid cultures and involve estimation of lipid content in yeasts using the expensive fluorescent dye Nile Red and a fluorimeter, and, as a result, these methods are limited by the rapid quenching of the fluorescence. This limitation causes notable variation in the experimental results, and many laboratories do not have access to this type of equipment.

Other quantitative screening methods are based on colorimetric or spectrophotometric techniques. These approaches are less expensive than the fluorometric ones because they only require a spectrophotometer, which is less expensive and more commonly available in most laboratories than a fluorimeter, but they still have the disadvantage of requiring liquid cultures, long sample processing times, and the usage of some toxic or dangerous compounds. Examples of such methods include those described by Thakur et al. [8], which involves the use of Sudan Black B, Shin et al. [9], which involves the use of Oil Red O, and Izard et al. [10], which is based on the sulfo-phospho-vanillin reaction with lipids. In contrast, qualitative methods for the screening of oleaginous microorganisms have also been reported. One of the most commonly used methods was described by Norris et al. [11], which involves the use of Sudan Black B to stain intracellular lipids black [12, 13] and safranin to counterstain the rest of the cell red. However, Norris' technique requires liquid cultures and additional steps such as smear preparation and microscope visualization.

Another recently reported technique for the screening of oleaginous yeasts necessitates the use of a Bodipy probe to directly view yeasts under the fluorescence microscope and thus observe the lipid droplets inside the cells [14]. This technique also has the drawback of requiring lipid cultures and is not adequate for the detection of weak lipid-producing yeasts.

Another widely used qualitative method for screening oleaginous yeasts is the one described by Evans et al. [15], which has the advantage of requiring the use of agar cultures. However, this method also requires multiple additional steps after agar incubation, such as printing a replica of the agar plate on filter paper, staining with Sudan Black B, washing with ethanol, and drying, and it is therefore time- and resource-consuming. In addition, there are risks of strain contamination during the replica printing step. Colonies with a high lipid content appear as dark blue/purple spots, whereas those with a low lipid either are colored sky blue or remain unstained.

The dye Rhodamine B has been used for staining oils in media in Petri dishes with the aim of determining microbial lipase activity [16]. However, prior to this study, this dye had not been used to stain intracellular lipids in microorganisms cultured on Petri dishes.

Based on the above-mentioned methods, the screening of oleaginous microorganisms among a large number of strains is very expensive and time-consuming due to the multiple steps involved. Therefore, the screening of a large number of wild-type strains or mutants requires a faster and more economical method. The aims of this work were to develop a rapid method for screening oleaginous yeasts, to identify the levels of lipid accumulation directly from agar cultures and to validate the developed method through the direct quantification of lipids in the colonies. Furthermore, the applicability of the method with other carbon sources was also studied.

## 2. Materials and Methods

### 2.1. Yeast Strains

The yeasts* Yarrowia lipolytica* ATCC 9773 and* Saccharomyces cerevisiae* Ethanol Red (Fermentis 42138) were previously categorized in our lab as oleaginous (more than 50% lipids) and non-oleaginous (less than 20% lipids), respectively, and were thus used as positive and negative controls, respectively.

Two reddish-pigmented yeasts, which were also isolated from soil in our lab, were used to test an alternative application of this method for these types of yeasts. Both reddish yeasts were identified as* Rhodotorula mucilaginosa* by PCR-RFLP according to Segura et al. [17] and are referred to as* R. mucilaginosa* A and* R. mucilaginosa* B.

Additionally, 124 recently isolated wild-type nonpigmented (white) yeast strains from our lab collection were used. These yeasts were isolated mainly from soil and food waste. Most of the yeasts have not yet been identified and are thus labeled in this work with consecutive numbers.

### 2.2. Media and Culture Conditions

Because a high carbon-to-nitrogen ratio (C/N) improves lipid accumulation, a nitrogen-limited medium was used to develop the method. This medium was based on the protocol described by Suutari et al. [18] and has the following composition: 23 g/L glucose, 0.3 g/L peptone, 0.5 g/L yeast extract, 7 g/L KH_2_PO_4_, 2 g/L Na_2_HPO_4_*∙*7H_2_O, 1.5 g/L MgSO_4_, and 20 g/L agar (pH 5.5±0.2), corresponding to a C/N of 80.

A rich medium (YPD) was also prepared with the following composition: 20 g/L glucose, 20 g/L peptone, 10 g/L yeast extract, and 20 g/L agar (pH 5.5±0.2), corresponding to a C/N of 2.1 and it is referred to as a low-C/N medium.

Two different media with alternative carbon sources were also tested. The first one had the same composition as the above-described nitrogen-limiting medium with the exception that pure glycerol was used instead of glucose. The second medium was prepared using agave bagasse hydrolysate as described before [19] and had the following composition: bagasse hydrolysate (80% v/v, which corresponds to 20 g/L glucose and 10 g/L xylose), phosphate buffer (50 mM, pH 6.8, 20% v/v), 1.7 g/L yeast nitrogen base, 1.5 g/L NH_4_Cl, and 20 g/L agar.

When indicated, dye solutions were added to all the media as described in the dye preparation section.

All yeasts were maintained in YPD agar plates and stored at 4°C for up to 3 months. Inoculums were prepared by taking isolated colonies from stored yeasts, which were plated on YPD and incubated at 30°C for 1 day. For media evaluation, isolated yeast colonies from fresh inoculums were taken and put as single points on the test plates. Afterwards, plates were incubated at 30°C for 2 days and examined regularly.

### 2.3. Dye Preparation

**Figure 1:**
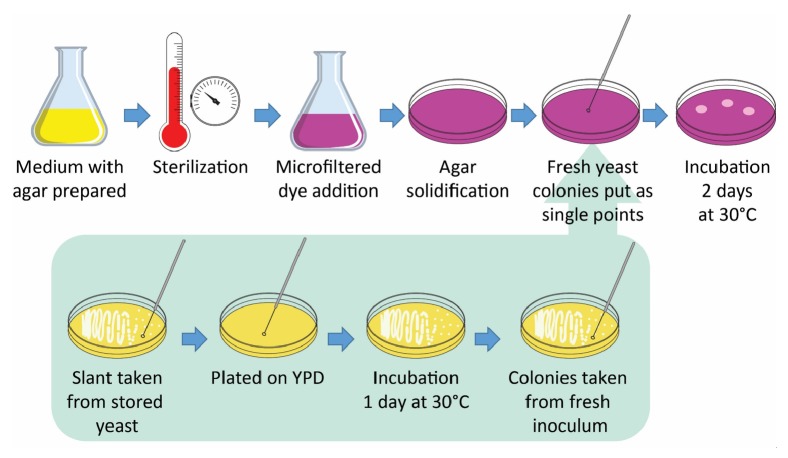
Diagram of the proposed procedure using the selected dye, Rhodamine B.

Different previously reported lipid dyes were tested: Oil Red O [9] (Sigma-Aldrich O0625), Sudan Black B [8, 15] (Sigma-Aldrich 199664), Nile Red [6] (Sigma-Aldrich N3013), and Rhodamine B [16, 20] (Sigma-Aldrich 83689). All the dyes were dissolved in their respective solvent, according to previous reports (see Table 1), and filtered through a 0.22-*μ*m sterile syringe membrane (Millex SLGS033SB). The microfiltered dye solutions were added to sterile agar media, and the resulting media were added to Petri dishes (similar to the process shown in Figure 1). Agar media were placed in sterile Petri dishes, and once the agar solidified, the plates were inoculated and incubated.

### 2.4. Fluorescent Examination

For fluorescent examination, a UV lamp (UVGL-15 Compact UV Lamp) at 365 nm was used, and the plates were examined in a dark room using appropriate personal protection equipment.

### 2.5. Lipid Content Quantification

To validate the proposed method, the qualitative results obtained were compared to the lipid content in three yeast colonies on each test plate. First, each colony was placed in preweighted 2-mL Eppendorf® tubes, and 1 mL of distilled water was added to each tube. The biomass was dispersed and frozen at -20°C. Once frozen, the tubes were lyophilized and then weighted to determine the dry cell weight (DCW) of each colony. The lipids from the freeze-dried biomass were then extracted using a procedure based on that described by Folch et al. [21]. Briefly, a chloroform:methanol mixture (2:1, v/v) was added, and the samples were sonicated and centrifuged. Finally, lipid extracts were recovered by evaporation of the bottom layer solvent. The obtained lipids were weighed, and the lipid percentage content of each sample was calculated in terms of the DCW.

## 3. Results and Discussion

### 3.1. Dye Selection

The first step in the development of the method was to use different dyes and preparation procedures using both rich and nitrogen-limited media based on the reference methods listed in Table 1. The dye solutions were microfiltered to avoid heat-induced degradation and added after medium sterilization but prior to solidification. Once the agar solidified, the plates were inoculated and incubated for 2 days at 30°C.

All plates were observed under visible light. The objective was to select the dye that presented the most easily detectable response to lipid accumulation. The expected response was establishment of a clear difference in the colony color between the oleaginous and non-oleaginous yeasts studied. Among the studied dyes, only Sudan Black B and Rhodamine B showed differences between the oleaginous and non-oleaginous yeasts. However, Rhodamine B showed the best differentiation and was thus selected as the optimal dye for the developed method. In addition, Rhodamine B has the advantages of requiring an easier and cheaper preparation than Sudan Black B (Table 1).

The selected dye, Rhodamine B (C_28_H_31_N_2_O_3_Cl; mol. wt. 479; IUPAC name N-[9-(ortho-carboxyphenyl)-6-(diethylamino)-3H-xanthen-3-ylidene] diethyl ammonium chloride), is a highly water soluble, basic red dye of the xanthene class. This dye has been used to stain lipids in different applications [16, 20, 22, 23]. At the prepared concentrations used in this work and under the proposed conditions, this dye is neither a pollutant nor a hazardous substance.

**Figure 2:**
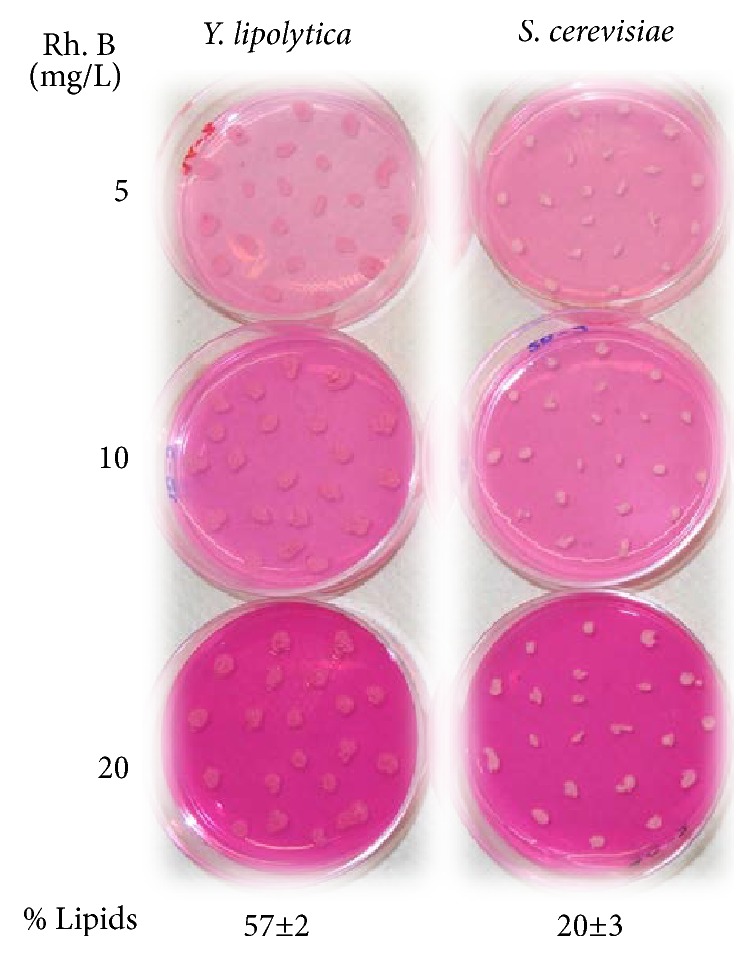
*Y. lipolytica* and* S. cerevisiae* were cultured on N-limited medium at three different concentrations of Rhodamine B (Rh. B) for two incubation days. The lipid content was measured in triplicate.

Using Rhodamine B at 10 mg/L [16],* Y. lipolytica* colonies appeared in an intense pink color, whereas* S. cerevisiae* colonies were colored white after two incubation days, as shown in Figure 2. Because both yeasts grow as white colonies on dye-free media, this result indicates that Rhodamine B was able to penetrate the cell membranes and stain the lipid droplets inside the cells.

A diagram of the final procedure with the selected dye is presented in Figure 1. It is worth mentioning that shielding the dye solution from light is not necessary because the same results were obtained under dark and light conditions.

### 3.2. Rhodamine B Concentration Selection

To check whether a Rhodamine B concentration of 10 mg/L was optimal, different Rhodamine B concentrations, namely, 5, 10, and 20 mg/L (Table 1), were used with the oleaginous and non-oleaginous model yeasts.

As shown in Figure 2, color differentiation between the two types of yeasts can be observed with all Rhodamine B concentrations. The yeast* Y. lipolytica* appears in an intense pink color, which is similar to the medium color. However, 10 mg/L Rhodamine B resulted in more evident differentiation between oleaginous and non-oleaginous yeasts. This differentiation corresponds to lipid percentages of 57±2% in* Y. lipolytica* and 20±3% in* S. cerevisiae*, respectively. At a Rhodamine B concentration of 20 mg/L, color differentiation was also evident, but some of the color was also due to the yeast* S. cerevisiae*. Given all the above-described findings, 10 mg/L was selected as the optimal concentration.

### 3.3. Carbon-to-Nitrogen Ratio (C/N) Selection

As previously mentioned, high-C/N values can induce lipid accumulation in microorganisms. To assess whether the C/N of 80 used in previous experiments was optimal for the developed method, three different high-C/N media and a low-C/N (or rich) medium were tested with the oleaginous yeast* Y. lipolytica*. The C/N values analyzed corresponded to 140, 80, 40, and 2.1. These values were based on the media described in Materials and Methods with the exception that the glucose concentration was changed for the media C/N values of 140 and 40.

**Figure 3:**
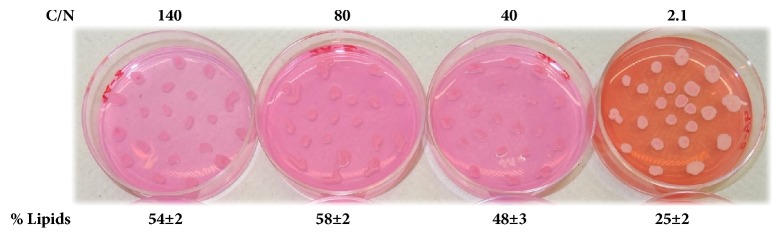
*Y. lipolytica* was cultured media with different C/N values (140, 80, 40, and 2.1) with 10 mg/L Rhodamine B for two incubation days. The lipid content was measured in triplicate.

As expected, the pink color of the colonies in the rich (low-C/N) medium was less intense, whereas all the colonies on high-C/N media turned into an intense pink color because more lipids are produced with increasing C/N (Figure 3). Indeed, similar results were obtained after lipid quantification. In fact, among the C/N values tested, the optimal ratio for lipid accumulation was 80, which corresponded to a lipid content of 58±2%. Therefore, our method can also be used to select the best C/N in terms of increased lipid production.

### 3.4. Response Levels of the Method

As mentioned above, the response obtained using the qualitative method is the pink color of the yeast colonies, which are white or yellowish in media in the absence of dye. To verify that a higher pink color intensity corresponds to a higher lipid content, this parameter was measured and related to the color intensity of different yeasts.

**Figure 4:**
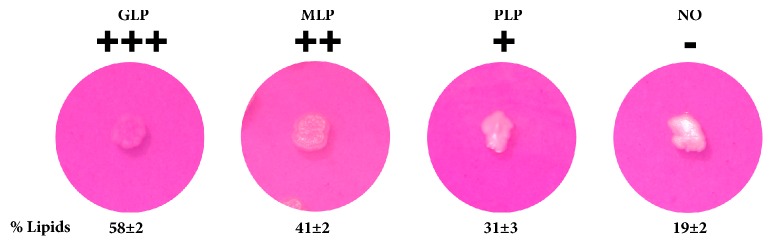
Qualitative response levels of the screening method for oleaginous yeasts. A close-up of single colonies is shown. The levels are defined as follows: GLP, good lipid producer; MLP, moderate lipid producer; PLP, poor lipid producer; and NO, non-oleaginous. The lipid percentage is presented as % of lipids, and the data are presented as the means and standard deviations from triplicate samples.

The lipid accumulation level for screening purposes was defined as the pink intensity of the grown colonies. The different symbols in Figure 4 indicate the different levels of lipid accumulation and color intensity: the symbol “-” indicates non-oleaginous yeasts (abbreviated NO), which are strains that did not turn pink or showed a white or yellowish center, the symbol “+” denotes a slightly stained colony or poor lipid producer (abbreviated PLP), the symbol “++” indicates a moderate-stained colony or moderate lipid producer (abbreviated MLP), and the symbol “+++” designates an intense pink stained colony, which was almost the same color as the medium, or a good lipid producer (abbreviated GLP). These levels were correlated with the lipid contents of the yeasts.

It is worth mentioning that the culture time used in the method described by Evans [15] is 3 to 4 inoculation days on agar, approximately 4 h for sample processing (including the replica printing on filter paper) and an additional variable amount of time depending on the drying oven, resulting in a total culture time of approximately 5 to 6 days from inoculation to results. Using our Rhodamine B-based method, 2 days of culture on agar is sufficient, and no further processing steps are required.

### 3.5. Alternative Carbon Sources

Because the exploration of alternative carbon sources is of great interest to reduce costs, the developed method was also validated using two different substrates. The first alternative source tested was glycerol, and the tested medium was designed as described in Materials and Methods with glycerol instead of glucose. The second medium tested involved the use of lignocellulosic hydrolysate as mentioned in Materials and Methods. In both media, Rhodamine B was added at a concentration of 10 mg/L.

**Figure 5:**
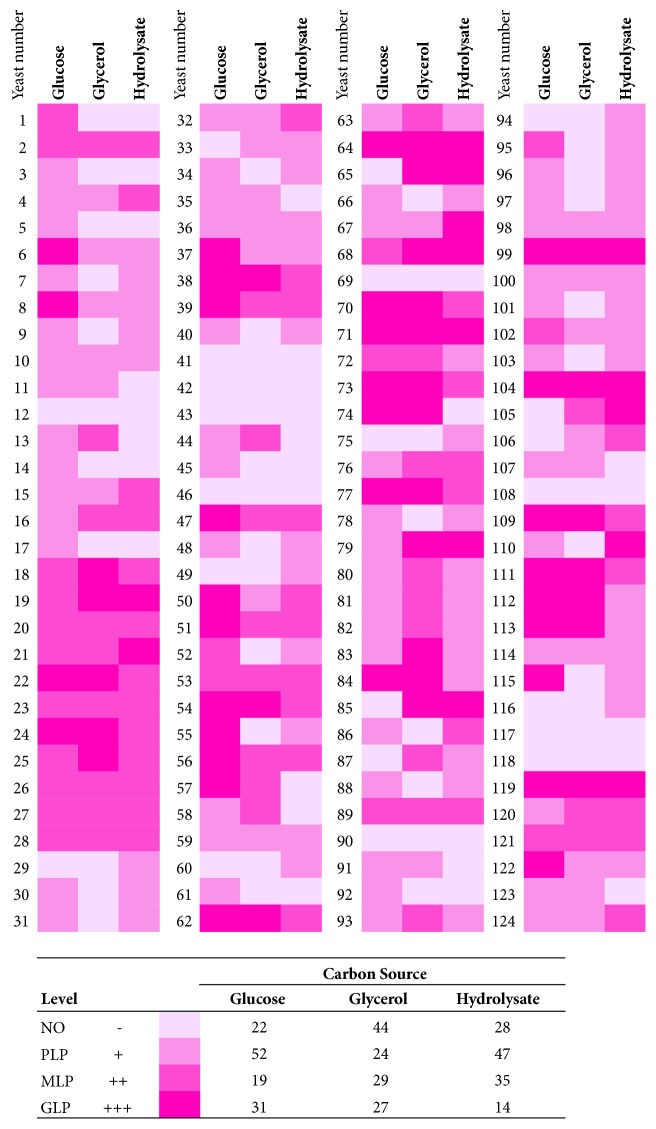
Screening of oleaginous white yeasts on different carbon sources: glucose, glycerol, and lignocellulosic hydrolysate. The yeasts were classified as follows: GLP, good lipid producer; MLP, moderate lipid producer; PLP, poor lipid producer; and NO, non-oleaginous. A total of 372 (124×3) plates (size 35×10 mm) were used in this experiment.

The same lipid contents were obtained using the alternative carbon sources. The developed media were used for the screening of oleaginous strains from 124 recently isolated wild-type nonpigmented yeast strains in our lab collection. The strains were screened for their lipid production capacity over a 48-h incubation period. Using the qualitative response levels described above (Figure 4), the strains were classified after the incubation time as shown in Figure 5. The results obtained with a traditional carbon source, such as glucose, show that 31 strains were GLPs. In contrast, using glycerol, 27 strains were identified as GLPs, whereas in the media with lignocellulosic hydrolysate, only 14 yeasts showed good lipid accumulation.

Few strains were identified as GLP in the medium with lignocellulosic hydrolysate because, similarly to all lignocellulosic residues, this medium contains xylose and inhibitory compounds [19]. Not all strains are capable of assimilating this type of sugar in the presence of these compounds [19, 24, 25], which limits the number of strains capable of using lignocellulosic materials. This variation of the method can also be applied to other lignocellulosic residues to reduce the time and resources usually used when selecting oleaginous yeasts [25, 26]. This method also allows the selection of oleaginous yeasts by evaluating two important aspects at the same time: growth on lignocellulosic residues and lipid production capacity.

As shown in Figure 5, five yeasts were classified as GLP in all three media (strains 64, 71, 99, 104, and 119), reflecting their ability to use the three different carbon sources tested in this study. Selected colonies were compared to the reference oleaginous yeast (*Y. lipolytica*), and their lipid contents were higher than 48%. Specifically, the lipid contents in strains 64, 71, 99, 104, and 119 were 48, 49, 52, 55, and 53%, respectively. Although strains are not fully identified, some oleaginous yeast genera found in addition to* Yarrowia* were* Candida* and* Debaryomyces*.

It is worth mentioning that the same responses were obtained when the agar plates with Rhodamine B were stored at 4°C for more than two months prior to processing, which indicates that the proposed method and its alternatives show good stability.

### 3.6. Adaptation of the Screening Method for Reddish-Pigmented Yeasts

Similar to Evans' technique [15], the new method is only useful for nonpigmented yeasts. Indeed, the color of the reddish-pigmented yeasts interferes with the responses obtained using this method under visible light.

Therefore, this assay was tested with reddish-pigmented yeasts, such as* Rhodotorula mucilaginosa*, using a different approach. Because Rhodamine B is also a fluorophore under UV light [16], the fluorescence of colonies dyed with Rhodamine B was also observed. As shown in Figure 3, the rich medium (low-C/N or YPD) plate looks more reddish than the plates with the high-C/N media, and this difference might be due to the peptone and yeast extract content of the medium. This darker background allowed a better contrast between the different colonies in the rich medium plates under UV light. To promote lipid accumulation in this low-C/N medium, the yeasts were incubated for up to four days to exhaust the available nutrients and allow lipid accumulation.

**Figure 6:**
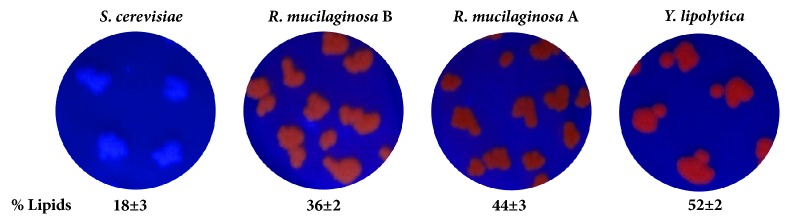
Visualization of the strains under UV light after incubation in rich medium (YPD) with 10 mg/L Rhodamine B for up to four days. The lipid content was measured in triplicate.

As expected, based on the orange fluorescence intensity (Figure 6), it was easy to differentiate the non-oleaginous yeast (*S. cerevisiae*) from the oleaginous strains, including the reddish-pigmented* R. mucilaginosa* A and B. Furthermore, this method allowed the assessment of the* R. mucilaginosa* strain—A or B—that produced more lipids. The screening method showed that* R. mucilaginosa* B was more oleaginous, and this result was verified by its lipid percentage (see lipid percentages in Figure 6).* Y. lipolytica* had the highest lipid content and showed a higher orange fluorescence.

### 3.7. Summary of the Protocol for the Assay for White or Nonpigmented Yeasts

1. Prepare the agar medium with the following model composition: 23 g/L glucose, 0.3 g/L peptone, 0.5 g/L yeast extract, 7 g/L KH_2_PO_4_, 2 g/L Na_2_HPO_4_*∙*7H_2_O, 1.5 g/L MgSO_4_, and 20 g/L agar (pH 5.5±0.2). Other carbon sources can be used by adapting their concentration to the equivalent amount of glucose.
2. Sterilize the Medium
3. Add a microfiltered Rhodamine B solution with a concentration of 1 g/L in acetone at a dose of 10 mL/L to obtain a final concentration of 10 mg/L in the medium.
4. Pour the medium into Petri dishes and allow it to solidify.
5. Inoculate the plate with fresh yeast colonies.
6. Incubate the plate at 30°C for 2 days.
7. Evaluate the yeast colony color according to Figure 4.

### 3.8. Summary of the Protocol for the Assay for Red or Reddish-Pigmented Yeasts

1. Prepare the agar medium with the following model composition: 20 g/L glucose, 20 g/L peptone, 10 g/L yeast extract, and 20 g/L agar (pH 5.5±0.2). This composition was selected to obtain a dark background in the plates.
2. Sterilize the medium.
3. Add a microfiltered Rhodamine B solution with a concentration of 1 g/L in acetone at a dose of 10 mL/L to obtain a final concentration of 10 mg/L in the medium.
4. Pour the medium into Petri dishes and allow it to solidify.
5. Inoculate the plate with fresh yeast colonies.
6. Incubate the plate at 30°C for 4 days.
7. Evaluate the yeast colony color according to Figure 6.

## 4. Conclusions

The developed qualitative method for screening oleaginous yeasts is a suitable option for screening a large number of strains and can be adapted for the analysis of reddish-pigmented yeasts. In addition, it can also be used when several carbon sources or nutrients are of interest. These procedures are less time- and resource-consuming because they do not depend on measurements, liquid cultures, or complicated equipment. The response is obtained directly from colonies grown on agar, and their lipid production capacity can be classified simply by visualizing the plates under visible or UV light. To the best of our knowledge, the proposed alternative for screening oleaginous strains in reddish-pigmented yeasts constitutes the first report of a qualitative procedure for these types of microorganisms. Furthermore, the developed method and its alternatives can be applied to media improvement, high-throughput screening in automatized systems, and food technology, indicating its high versatility. Additionally, the extension of the assay to oleaginous fungi and bacteria is also being explored.

## Figures and Tables

**Table 1:** Lipid dyes tested for the screening of oleaginous yeasts.

Dye	Solvent	Stock solution concentration (g/L)	Dye concentration in media	Price*∗* (USD/L culture)	References
Oil Red O	Isopropanol	30	9 mL/mL	230	[9]
Sudan Black B	Ethanol	30	2.4 g/L	7	[8]
Nile Red	Acetone	0.1	10 mg/L	0.5	[6]
Rhodamine B	Ethanol	1	10 mg/L	0.004	[16], this work
Rhodamine B	Ethanol	1	5 mg/L	0.002	This work
Rhodamine B	Ethanol	1	20 mg/L	0.008	This work

*∗*Calculated from prices in Sigma-Aldrich (http://www.sigmaaldrich.com/).

## Data Availability

All data generated or analyzed during this study are included in this article.
